# Molecular Testing Among Patients With Advanced Non–Small Cell Lung Cancer in the Rural US

**DOI:** 10.1001/jamanetworkopen.2026.42270

**Published:** 2026-11-05

**Authors:** Dena P. Rhinehart, Evelien van Gelderen, Josephine Feliciano, Craig Evan Pollack, Kristen A. Marrone, Amanda L. Blackford

**Affiliations:** 1 Department of Oncology, The Sidney Kimmel Comprehensive Cancer Center at Johns Hopkins University, Baltimore, Maryland; 2 School of Medicine, Johns Hopkins University, Baltimore, Maryland; 3 Johns Hopkins Bloomberg School of Public Health, Johns Hopkins University, Baltimore, Maryland; 4 Johns Hopkins School of Nursing, Johns Hopkins University, Baltimore, Maryland

## Abstract

**Importance:**

Molecular evaluation is standard of care for patients with advanced non–small cell lung cancer (NSCLC) in the US. Patients in rural areas face disproportionate lung cancer mortality associated with reduced guideline-concordant evaluation and treatment receipt, but molecular testing patterns have not been described in this population.

**Objective:**

To characterize molecular testing patterns in the rural US following initial testing implementation.

**Design, Setting, and Participants:**

This cohort study used the Surveillance, Epidemiology, and End Results Program linked to Medicare claims data to identify individuals aged 66 years or older diagnosed with advanced NSCLC from January 2014 to December 2019. Data analysis was performed from December 2025 to August 2026.

**Exposures:**

Sociodemographic variables, histology, and practitioner visits.

**Main Outcomes and Measures:**

The primary outcome was receipt of molecular testing. The cumulative incidence of testing overall and by patient subgroups defined at baseline were calculated using the total person-time at risk (30 days before diagnosis to death or 6 months after diagnosis) using a competing-risks framework, with death as the competing event.

**Results:**

Among a total of 8259 Medicare beneficiaries residing in rural areas who were diagnosed with advanced NSCLC included in the study (mean [SD] age, 76.1 [6.7] years; 4672 males [56.6%]), 1775 (21.5%) were eligible for Medicaid, 3754 (45.5%) had adenocarcinoma histology, 2044 (24.7%) had squamous histology, and 2461 (29.8%) had other or unspecified histology. In the cohort, 2963 (35.9%) received molecular testing. The cumulative incidence of testing by 180 days after diagnosis was 0.36 (95% CI, 0.35-0.37). Increasing age (hazard ratio [HR], 0.96 [95% CI, 0.93-0.99]), later diagnosis year (HR, 1.05 [95% CI, 1.02-1.07]), dual Medicaid coverage (HR, 0.71 [95% CI, 0.64-0.79]), Part D enrollment (HR, 1.13 [95% CI, 1.04-1.22]), and squamous histology (HR, 0.35 [95% CI, 0.32-0.39]) were independently associated with testing. A greater percentage of patients who received testing had visits with both a proceduralist or a surgeon and an oncologist compared with those without testing (1257 [77.7%] vs 867 [58.9%]; *P* < .001).

**Conclusions and Relevance:**

In this cohort study of Medicare beneficiaries with advanced NSCLC residing in the rural US, initial receipt of molecular testing was low. Dual Medicaid coverage, Part D enrollment, and subspecialist visits were independently associated with test receipt, highlighting key areas for future study to promote equitable delivery of guideline-concordant lung cancer care.

## Key Points

**Question:**

What factors are associated with molecular testing among older adults with advanced non–small cell lung cancer in the rural US after guideline adoption?

**Findings:**

In this cohort study of 8259 patients with advanced non–small cell lung cancer in the rural US from 2014 to 2019, less than 40% of patients received testing. Age, diagnosis year, histology, dual Medicaid and Medicare enrollment, Part D enrollment, and subspecialist involvement were independently associated with test receipt.

**Meaning:**

The findings of this study suggest that testing receipt was low in the rural US, underscoring the need for additional study to examine testing equity as molecularly informed care expands.

## Teaser

This cohort study examines factors associated with molecular testing among older adults with advanced non–small cell lung cancer (NSCLC) in the rural US.

## Introduction

Despite improvements in lung cancer survival in the US, there is a growing rural-urban mortality gap.^1,2,3^ Not only do environmental, occupational, and smoking exposures contribute to a higher incidence of lung cancer among rural populations, but also, once diagnosed, patients who reside in rural areas have reduced access to treatment advances and are less likely to receive treatment for metastatic disease.^1,3,4^ When patients from rural areas with advanced non–small cell lung cancer (NSCLC) are provided the same treatment as those who reside in urban areas, overall survival is comparable.^5^

Access to guideline-concordant care is a key modifiable risk factor in rural-urban survival disparities. A critical aspect of NSCLC care is the use of molecular testing to guide targeted therapies. Since its introduction in 2011^6^ and 2013,^7^ targeted therapy has been associated with improved survival compared with platinum-based chemotherapy.^8^ Accordingly, molecular testing to identify patients who are eligible for these therapies has been recommended by national and international guidelines since 2013^9^ and is a standard-of-care evaluation.^10^ In 2018, the Centers for Medicare & Medicaid Services issued a national coverage determination for Food and Drug Administration (FDA)–approved next-generation sequencing tests to assess patient eligibility for targeted therapies.^11^ Since that time, targeted therapy indications have expanded rapidly across NSCLC, with 5 FDA approvals in 2025 alone.^12^

Despite the clinical relevance of molecular testing to guide therapy, use among rural populations has been suboptimal, with rural populations having 30% to 40% lower odds of receiving molecular testing.^13,14^ There are several likely mechanisms explaining reduced uptake of molecular testing among rural populations. First, targeted therapy use is highest at resource-rich National Cancer Institute–designated and academic centers,^15^ which are concentrated in urban areas.^16^ These centers typically have integrated multidisciplinary specialists and advanced pathology resources, which facilitate a traditional pathway in which proceduralists obtain tumor tissue; then medical oncologists order molecular testing^17,18^ and deliver systemic treatment in the same health system. In contrast, rural health care is particularly at risk for workforce shortages,^19^ fragmented care, and limited infrastructure, which may challenge this delivery strategy. In addition, several patient-level factors associated with reduced testing or treatment rates, such as older age, dual Medicaid enrollment, greater comorbidity burden, and nonadenocarcinoma histology, are more prevalent in rural areas.^4,13,14,15^

In this study, we first examined sociodemographic and clinical factors associated with receipt of molecular testing between 2014 and 2019, a key time for adoption of these technologies, among fee-for-service Medicare beneficiaries with NSCLC residing in rural areas. We then examined the association between subspecialist involvement and testing and whether patients who received molecular testing were more likely to receive systemic therapy. Elucidating factors historically associated with receipt of molecular testing and systemic treatment can help guide further study of contemporary testing patterns and inform studies to expand access to guideline-concordant NSCLC care among older adults in rural areas.

## Methods

### Data Source and Study Population

In this cohort study, we identified a cohort of patients diagnosed with advanced NSCLC as their first primary cancer between January 2014 and December 2019 using the Surveillance, Epidemiology, and End Results (SEER) cancer registry program and Medicare-linked database. Those with rural residence at diagnosis were included, with rurality defined using county-level Rural-Urban Continuum Codes 4 through 9 in accordance with the National Cancer Institute classification. NSCLC diagnosis was identified by Site Recode/World Health Organization 2008 *International Classification of Diseases, 3rd Edition for Oncology* (*ICD-O-3*) codes 22030 and histology codes (eTable 1 in Supplement 1). Distant stage at diagnosis was identified using the SEER Combined Summary Staging, as reported in the SEER-Medicare database. Individuals, aged 66 to 95 years at diagnosis and continuously enrolled in fee-for-service Medicare Parts A and B for at least 12 months before diagnosis and through death or 6 months after diagnosis, were included. Those diagnosed by autopsy or death certificate were excluded. This study was reviewed by the Johns Hopkins Institutional Review Board as exempt and not requiring informed consent because it used deidentified data. This study followed the Strengthening the Reporting of Observational Studies in Epidemiology (STROBE) reporting guideline for cohort studies.

### Outcomes

The primary outcome was the time to receipt of molecular testing, as identified through inpatient, outpatient, and physician carrier claims using the Healthcare Common Procedure Coding System codes (see eTable 2 in Supplement 1). Codes were derived from prior molecular testing studies^14,15,20^ including single gene mutation assays (ie, epidermal growth factor receptor), multigene sequencing methods (ie, next-generation sequencing), fluorescence in situ hybridization (ie, for anaplastic lymphoma kinase fusions), protein assays, and institutional proprietary genomic panels. Claims for immunohistochemistry (IHC) were not included, as guidelines at the time did not recommend this method for selection of targeted therapies.^9^ Individuals with a first claim for molecular testing 30 to 365 days before or 6 months after diagnosis were excluded, as this may have reflected testing for a separate malignant neoplasm.

The secondary outcomes included systemic therapy receipt among those continuously enrolled in Part D for 6 months, as many targeted therapies are covered under Part D. Receipt of systemic therapy included any chemotherapy and/or immunotherapy, oral targeted therapy (eTable 3 in Supplement 1), or both within 6 months of diagnosis, as identified through the National Drug Code Directory.

### Covariates

Histology was classified into 3 categories based on *ICD-O-3 *codes: adenocarcinoma histology, squamous histology, or other or unspecified histology, as defined in eTable 1 in Supplement 1. Demographic characteristics, including age at diagnosis, sex, race and ethnicity (defined by the SEER program using medical records), diagnosis year, marital status, the SEER registry, original reason for Medicare entitlement, and area-level Yost state-based quintile (a measure of socioeconomic status, in which scores range from 1 to 5, with 5 indicating higher socioeconomic status), were classified by and captured in the SEER file. Race and ethnicity categories included Hispanic, non-Hispanic American Indian or Alaska Native or Native Hawaiian or Other Pacific Islander, non-Hispanic Black, and non-Hispanic White. Race and ethnicity were included in the study as a measure of a social determinant of health. Medicaid and Part D enrollment were identified in the Master Beneficiary Summary File. The Charlson Comorbidity Index score, at least 1 inpatient hospitalization, and frailty score (dichotomized as less than 0.15 and 0.15 or greater, with higher scores indicating more frailty)^21^ were calculated in the year before diagnosis using inpatient, outpatient, and/or physician carrier claims. Visits with oncologists, proceduralists (including pulmonology or interventional radiology), or surgeons (including general surgeons and surgical subspecialists) were identified via claims associated with outpatient and carrier codes by specialty practitioner status (eTable 4 in Supplement 1) within 6 months of diagnosis.

### Statistical Analysis

Characteristics at baseline for the full cohort and among those who survived 6 months after diagnosis with continuous Part D coverage were summarized descriptively. The primary outcome, time to receipt of molecular testing, was analyzed using the full study cohort. The cumulative incidence of testing overall and by patient subgroups defined at baseline were calculated using the total person-time at risk (30 days before diagnosis to death or 6 months after diagnosis) using a competing-risks framework, with death as the competing event.^22,23^ Individuals who did not survive the first 6 months contributed their person-time from diagnosis to death. Hazard ratios (HRs) for differences in time to testing between groups were estimated using cause-specific Cox proportional hazards regression models.^24^

To identify a set of variables independently associated with testing, we used a group least absolute shrinkage and selection operator (LASSO) survival analysis. Group LASSO extends traditional LASSO by ensuring that all levels of a categorical variable are selected or excluded together, preventing the selection of individual factor levels in isolation.^25^ Candidate exposures included variables measured at baseline (diagnosis): age (continuous), sex, race and ethnicity, year of diagnosis, marital status, SEER region, Medicare entitlement reason, Medicaid enrollment status in the month of diagnosis, Yost state quintile, Charlson Comorbidity Index score, frailty score, hospitalization in the year before diagnosis, histology, and Part D enrollment status in the month of diagnosis. The penalty parameter was selected via 10-fold cross-validation on the full dataset to minimize a cross-validation error. We used bootstrap resampling with 1000 iterations to assess variable selection stability and construct CIs. In each bootstrap sample, we fit the group LASSO model using the preselected lambda parameter and recorded which covariates were selected and their coefficient estimates. Covariates selected in more than 80% of bootstrap samples were included in the final model, and percentile-based 95% CIs were calculated.

Sensitivity analyses included estimating the associations between patient characteristics and time to testing among the patients with adenocarcinoma histology only. Because of the temporal nature of practitioner specialty involvement after diagnosis, we examined its association with molecular testing in a subcohort of patients who survived 6 months after diagnosis. Logistic regression models with receipt of testing as a function of practitioner seen over 6 months (oncologist only, proceduralist or surgeon only, both, or neither) were estimated, adjusting for confounders.

The associations between the secondary outcomes (receipt of chemotherapy, immunotherapy, and oral-targeted therapy use) with receipt of molecular testing were estimated with logistic regression models, adjusted for confounders, in the subcohort of patients who survived 6 months and were continuously enrolled in Part D coverage. Among patients who received targeted therapy and another systemic therapy, we characterized treatment sequencing, including the proportion who received chemotherapy or immunotherapy before targeted therapy, and the time elapsed between treatment initiations.

Analyses were completed with R, version 4.5.1 (R Project for Statistical Computing)^26^ from December 2025 to August 2026. Two-sided *P* values less than .05 were considered statistically significant. Nominal *P* values are presented for descriptive purposes.

## Results

### Cohort Characteristics

The cohort included 8259 individuals residing in a rural county at the time of NSCLC diagnosis from 2014 to 2019 (eFigure in Supplement 1). The mean (SD) age was 76.1 (6.7) years; 3587 (43.4%) were females, and 4672 (56.6%) were males; 268 participants (3.2%) were Hispanic, 115 (1.4%) non-Hispanic American Indian or Alaska Native or Native Hawaiian or Other Pacific Islander, 357 (4.3%) non-Hispanic Black, and 7519 (91.0%) non-Hispanic White. Among all participants, 1775 (21.5%) had dual Medicaid and Medicare enrollment, and 2073 (25.1%) were enrolled in Part D low-income cost sharing. In addition, 3754 (45.5%) had adenocarcinoma histology, 2044 (24.7%) had squamous histology, 2461 (29.8%) had other or unspecified histology, and 5709 (69.1%) survived at least 60 days from diagnosis (Table 1).

**Table 1.** Descriptive Summary of Characteristics Measured at Non–Small Cell Lung Cancer Diagnosis and Survival Time From Diagnosis Among the Study Cohort

Factor	Patients, No. (%) (N = 8259)
**Characteristic at diagnosis**	
Age, y	
Mean (SD)	76.1 (6.7)
Median (range)	75.1 (66.0-95.0)
Sex	
Female	3587 (43.4)
Male	4672 (56.6)
Race and ethnicity	
Hispanic (all races)	268 (3.2)
Non-Hispanic	
American Indian or Alaska Native or Native Hawaiian or Other Pacific Islander	115 (1.4)
Black	357 (4.3)
White	7519 (91.0)
Marital status	
Married or domestic partner	2792 (33.8)
Single, separated, divorced, or widowed	2515 (30.5)
Unknown	2952 (35.7)
SEER region	
Northeast	920 (11.1)
South	4668 (56.5)
Midwest	1045 (12.7)
West	1626 (19.7)
Year of diagnosis	
2014	1535 (18.6)
2015	1549 (18.8)
2016	1376 (16.7)
2017	1378 (16.7)
2018	1212 (14.7)
2019	1209 (14.6)
Histologic type	
Adenocarcinoma	3754 (45.5)
Squamous	2044 (24.7)
Other or not otherwise specified	2461 (29.8)
Any Medicaid (full or partial) eligibility at diagnosis	
No	6484 (78.5)
Full or partial	1775 (21.5)
Reason for Medicare eligibility	
Age and survivor insurance	6809 (82.4)
Disability and/or end-stage kidney disease	1450 (17.6)
Part D low-income cost sharing	
Full	1807 (21.9)
Partial	266 (3.2)
Not eligible	6186 (74.9)
Yost index state quintile^a^	
1	2227 (27.0)
2	3142 (38.0)
3	2131 (25.8)
4	646 (7.8)
5	113 (1.4)
Charlson Comorbidity Index score	
0	4635 (56.1)
1	2215 (26.8)
≥2	1409 (17.1)
Hospitalized in year before diagnosis	
No	6415 (77.7)
Yes	1844 (22.3)
Frailty score^b^	
<0.15	3929 (47.6)
≥0.15	4330 (52.4)
**Survival time from diagnosis, d**	
Death	
<60	2550 (30.9)
60-90	1013 (12.3)
>90-120	701 (8.5)
>120-180	905 (11.0)
Alive >180	3090 (37.4)

Abbreviation: SEER, Surveillance, Epidemiology, and End Results.

^a^ Scores range from 1 to 5, with 5 indicating higher socioeconomic status.

^b^ Scores dichotomized as less than 0.15 and 0.15 or greater, with higher scores indicating more frailty.

The subcohort of patients who survived 6 months after diagnosis included 3090 individuals (eFigure in Supplement 1). Among them, those with continuous Part D coverage included 2046 individuals (66.2%) with similar demographics to the full cohort (eTable 5 in Supplement 1).

### Molecular Testing Patterns

In the total cohort, 2963 (35.9%) received molecular testing. Overall, the cumulative incidence of testing by 180 days after NSCLC diagnosis was 0.36 (95% CI, 0.35-0.37). The cumulative incidence was 0.18 (95% CI, 0.17-0.19) at 30 days, 0.30 (95% CI, 0.29-0.31) at 60 days, and 0.34 (95% CI, 0.33-0.34) by 90 days. Among patients with adenocarcinoma only, the cumulative incidence was 0.56 (95% CI, 0.55-0.58) by 180 days (eTable 6 in Supplement 1).

Figure 1 illustrates the time to testing according to patient subgroups, including differences by diagnosis year (HR, 1.36 [95% CI, 1.26-1.46] for those diagnosed after 2016), histology (HR, 0.30 [95% CI, 0.27-0.34] for squamous), and dual Medicaid enrollment (HR, 0.57 [95% CI, 0.52-0.63]). A sensitivity analysis among patients with adenocarcinoma showed results that were of similar direction and magnitude compared with the full cohort, although some of these results were nonsignificant (eTable 6 in Supplement 1).

**Figure 1.**
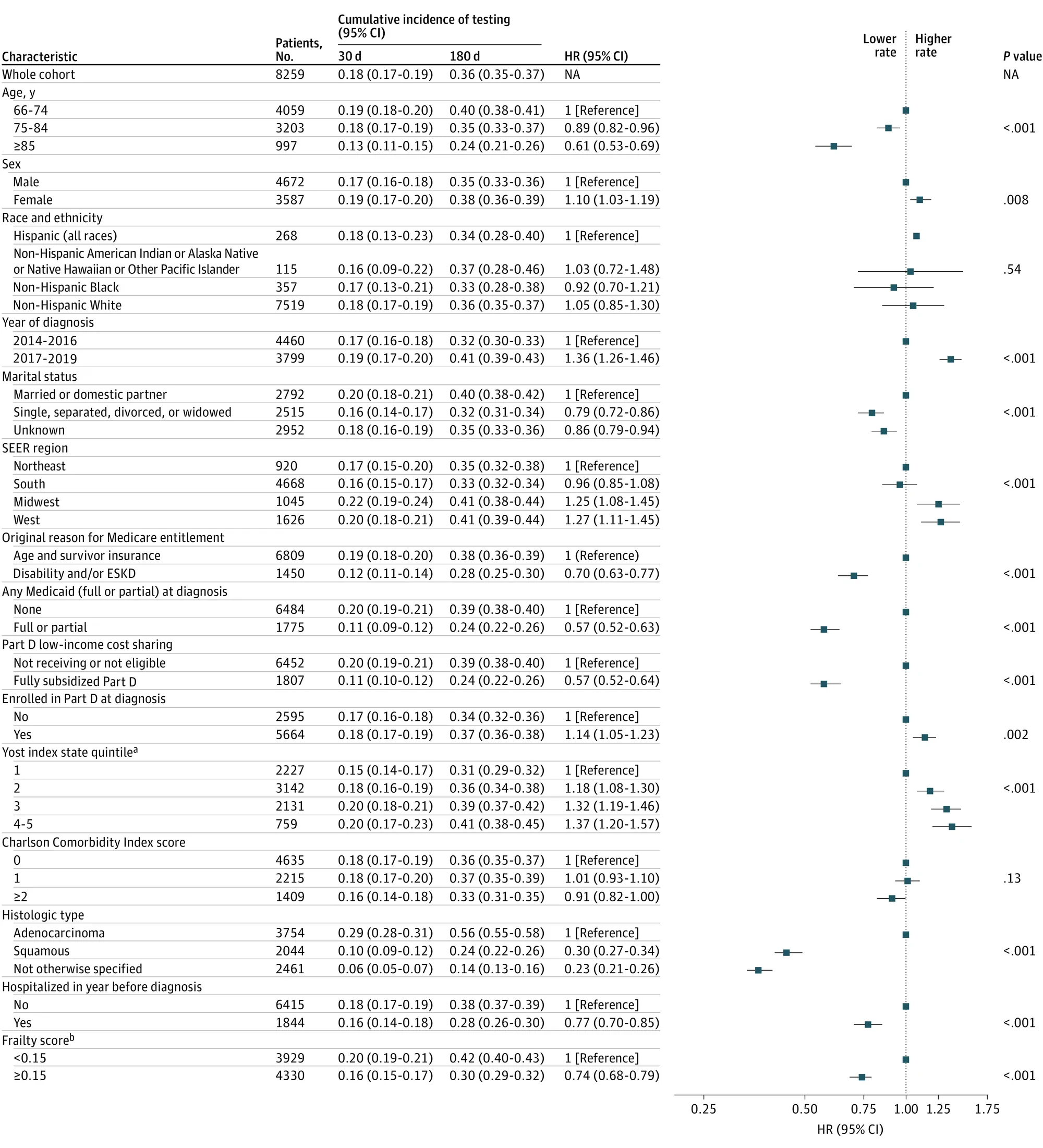
Dot Plot of Time to Molecular Testing Among Patient Subgroups Hazard ratios (HRs) for differences in time to testing among groups estimated from cause-specific Cox proportional hazards regression models. ESKD indicates end-stage kidney disease; NA, not applicable; SEER, Surveillance, Epidemiology, and End Results. ^a^Measure of socioeconomic status, in which scores range from 1 to 5, with higher scores indicating higher socioeconomic status. ^b^Dichotomized as less than 0.15 and 0.15 or greater, with higher scores indicating more frailty.

Figure 2 illustrates factors independently associated with testing on multivariable analysis. Patients with squamous histology (HR, 0.35 [95% CI, 0.32-0.39]), dual Medicaid enrollment (HR, 0.71 [95% CI, 0.64-0.79]), and increasing age (HR, 0.96 [95% CI, 0.93-0.99]) were less likely to be tested, while those with later diagnosis year (HR, 1.05 [95% CI, 1.02-1.07]) and Part D coverage (HR, 1.13 [95% CI, 1.04-1.22]) were more likely to be tested.

**Figure 2.**
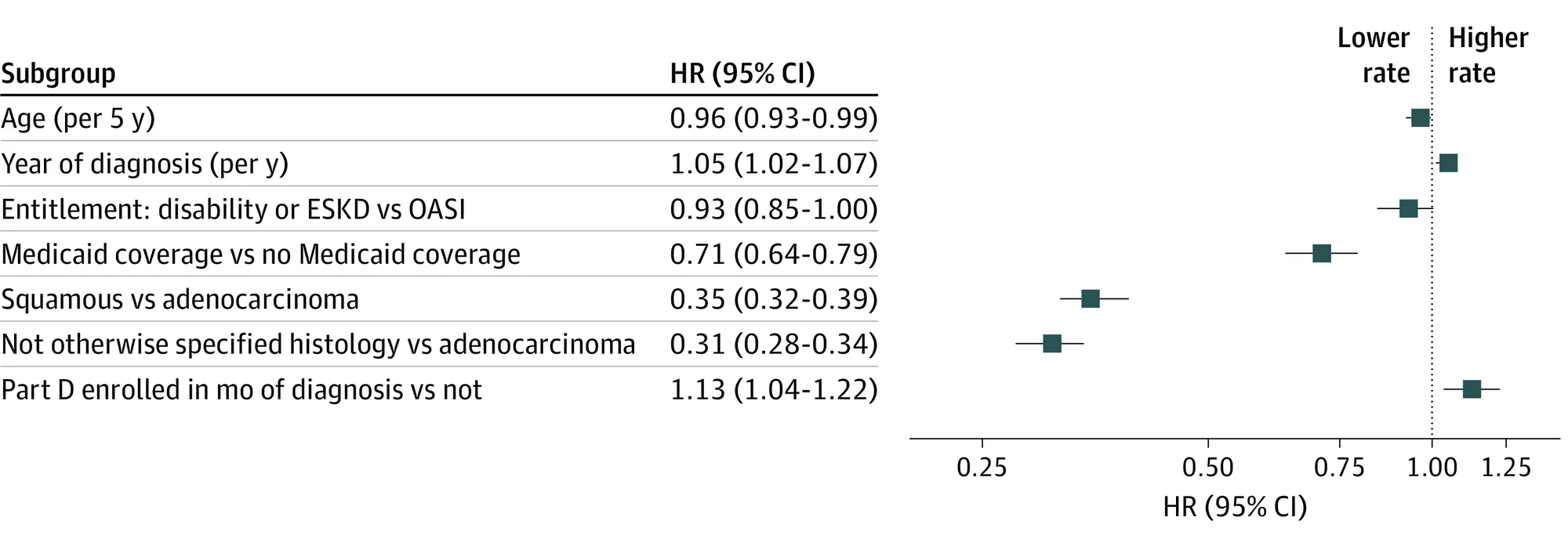
Dot Plot of Multivariable Analysis of Time to Molecular Testing Hazard ratios (HRs) for differences in time to testing among groups estimated from cause-specific Cox proportional hazards regression models. ESKD indicates end-stage kidney disease; OASI, Old-Age and Survivors Insurance.

### Molecular Testing, Subspecialty Care, and Systemic Therapy Receipt

Among the subcohort of patients surviving 6 months, subspecialist involvement varied by testing status (Table 2). Patients with testing were more likely to have seen both a proceduralist or a surgeon and an oncologist compared with those without testing (1257 [77.7%] vs 867 [58.9%]; *P* < .001).

**Table 2.** Frequency of Specialty Practitioner Visits According to Molecular Testing Receipt Among the Population Surviving at Least 6 Months

Practitioner specialty seen^a^	Patients, No. (%)		Odds ratio (95% CI)^b^
	Not tested (n = 1472)	Tested (n = 1618)	
Neither oncologist or proceduralist nor surgeon	196 (13.3)	20 (1.1)	1 [Reference]
Oncologist and proceduralist or surgeon	867 (58.9)	1257 (77.7)	14.45 (9.05-24.38)
Oncologist only	191 (13.0)	254 (15.7)	11.14 (6.74-19.25)
Proceduralist or surgeon only	218 (14.8)	87 (5.4)	3.97 (2.33-7.02)

^a^ Proceduralists included pulmonologists and interventional radiologists.

^b^ Logistic regression models for the receipt of molecular testing as a function of specialty practitioner mix seen, adjusted for diagnosis age, year of diagnosis, original reason for Medicare entitlement, enrollment in Medicaid in month of diagnosis, and adenocarcinoma histology.

Adjusted for factors independently associated with testing, among those who survived 6 months and had Part D coverage, those who were tested were more likely to receive systemic treatment, whether with chemotherapy and/or immunotherapy (odds ratio, 2.06 [95% CI, 1.64-2.60]; *P* < .001) or with targeted therapy (odds ratio, 3.75 [95% CI, 2.21-6.77]; *P* < .001), compared with those who were not tested (Table 3). Among the 136 patients who received targeted therapy, 58 (42.6%) also received chemotherapy and/or immunotherapy, with 32 of the 58 patients (55.2%) receiving chemotherapy and/or immunotherapy before targeted therapy (median [range], 28 [2-157] days).

**Table 3.** Frequency of Molecular Testing Receipt According to Receipt of Systemic Therapy Among Patients Surviving at Least 6 Months and Continuously Enrolled in Part D Coverage

Therapy type	Patients, No. (%)		Odds ratio (95% CI)^a^
	Did not receive	Received	
**Chemotherapy or immunotherapy**			
No. of patients	591	1455	NA
Receipt of testing			
Not tested	355 (60.1)	577 (39.7)	1 [Reference]
Tested	236 (39.9)	878 (60.3)	2.06 (1.64-2.60)
**Targeted therapy **			
No. of patients	1910	136	NA
Receipt of testing			
Not tested	916 (48.0)	16 (11.8)	1 [Reference]
Tested	994 (52.0)	120 (88.2)	3.75 (2.21-6.77)

Abbreviation: NA, not applicable.

^a^ Logistic regression models for the receipt of molecular testing as a function of therapy type, adjusted for diagnosis age, year of diagnosis, original reason for Medicare entitlement, enrollment in Medicaid in month of diagnosis, and adenocarcinoma histology.

## Discussion

While rural residence is a known risk factor for lower molecular testing and systemic therapy receipt, our findings identify which older adults in rural areas were more likely to experience gaps in testing during a key period for adoption of molecularly informed NSCLC care. While molecular testing implementation has evolved in recent years, these historical patterns may provide insight into specific rural populations at risk for undertesting and generate hypotheses for underlying mechanisms of testing disparities in the rural US health system.

Less than 40% of patients were tested within 180 days from diagnosis, with a modest increase in testing rates in more recent years, emphasizing persistent barriers. Advanced age, nonadenocarcinoma histology, and dual Medicare and Medicaid enrollment were negatively associated with test receipt, whereas subspecialist involvement at any time after diagnosis and Part D coverage were positively associated with testing. While molecular testing results were unknown and may have influenced treatment decisions, molecular testing itself was associated with higher rates of systemic treatment and may represent a marker of access to guideline-concordant care.^4^ In addition, over half (55.2%) of the patients who received targeted therapy had received chemotherapy prior, with a median interval of nearly 1 month, suggesting that testing delays may have contributed to suboptimal first-line treatment selection.

The low overall testing uptake, as well as associations between testing and therapy receipt, are consistent with previous reports. SEER-Medicare studies over similar periods have reported NSCLC molecular testing rates ranging from approximately 25% to 80%,^13,14,15,20^ although rates exceeding 50% were in studies that were restricted to patients who received systemic therapy.^15,20^ Studies examining rural-urban disparities reported approximately 30% to 40% lower odds of testing among rural vs urban subgroups.^13,14^ By assessing test receipt regardless of treatment status and long-term survival, we aimed to examine the potentially eligible population. Consistent with prior reports of the US population, we found that advanced age^13^ and dual Medicaid eligibility^13,14,15^ were independently associated with reduced testing in a rural-specific cohort. We added that Part D enrollment and subspecialist visits were associated with test receipt, and together, these findings suggest that access to testing may have varied by an individual’s resources to access and to navigate complex multidisciplinary care pathways.

We also examined all NSCLC histology, given both the evolving testing landscape and because nonadenocarcinoma histology is especially prevalent in rural populations. While patients with nonadenocarcinoma histology were less likely to be tested, testing was still less than 60% (56%) among those with adenocarcinoma, and similar disparities were seen in both groups. The difference in testing by histology was expected, as initial guidelines emphasized adenocarcinoma histology.^9^ However, as more recent literature has identified actionable targets in up to 40% of patients with a nonadenocarcinoma NSCLC histology,^27,28,29,30^ these findings may provide a historical benchmark as testing guidelines expand.^10^

While these patterns do not imply causality, they provide insight into possible mechanisms of testing disparities in the rural US health system. The association between dual Medicare and Medicaid enrollment and testing is likely quite complex. While dual enrollment may be a marker of socioeconomic vulnerability among older Medicare beneficiaries,^31,32,33^ it is also associated with limited access to ambulatory subspecialty care^34,35^ and greater likelihood of receiving care in structurally disinvested areas.^34,35,36^ Even if Medicare covers the cost of testing, dual-eligible patients are known to face disproportionate challenges (eg, less reliable transportation, less social support),^33^ which may be particularly relevant when navigating an NSCLC diagnosis in rural areas, in which specialist shortages are common,^19^ and distances are greater from comprehensive cancer centers^16^ with integrated multidisciplinary care and advanced pathology resources.

While we were unable to assess whether practitioner visits were associated with molecular testing, the pattern of subspecialist involvement with test receipt suggests that this may be a marker of access to multidisciplinary care, and supporting coordinated care pathways may help facilitate testing. For example, reflex molecular testing, whereby a diagnosis of NSCLC automatically triggers molecular testing, has been shown to reduce reliance on clinician-initiated testing^37^ and could help reduce gaps or delays as patients transition between clinicians.^17^ In the absence of reflex testing, workflow interventions such as electronic health record reminders and electronically integrated ordering, tracking, and reporting systems may also alleviate strain^38,39,40^ in busy practices.

Health system and policy interventions could also help expand access to testing and targeted therapies by leveraging centralized resources^41,42^ and funding shared regional molecular infrastructure, for example, modeled after the Health Resources & Services Administration’s Regional Genetics Networks.^43^ However, even if testing were accessible, downstream drug availability can be a barrier^44^ in rural settings, which may be reflected in the positive association between Part D coverage and testing in this cohort. However, even among those with Part D (which typically covers oral targeted therapy), there was a relatively low^45^ proportion of tested patients who received targeted therapy, which raises the possibility that not all eligible patients could access these drugs. Thus, examining barriers to targeted therapy in this population, such as cost and authorization burden,^46^ are areas for further investigation.

### Limitations

This study has several limitations. First, this is a study among older adults at a key time after the adoption of molecular testing. Testing techniques and implementation have evolved since the study period, and further study is needed to measure these disparities across additional years, patient ages, and settings. Second, while testing codes were derived from existing literature, there is potential for misclassification using claims data. Testing may be underestimated, for example, in the case of commercial non–FDA-approved tests, which may have contributed to the observed discordance between testing status and targeted therapy receipt. IHC tests (including programmed cell death 1 ligand 1) were not included, as these could not be differentiated from tissue-of-origin IHC tests over the study period. Third, these analyses are unable to present a causal relationship. There may be unmeasured confounding by other factors, such as tissue adequacy, patient treatment eligibility (eg, performance status), or care preferences (eg, decision to forgo systemic therapy). We also used county-level Rural-Urban Continuum Codes to define rurality, which is in line with governmental guidance but does not capture the heterogeneity of individual communities. Secondary analyses among those surviving 6 months may not be generalizable to the full cohort. In addition, we did not assess temporal associations between subspecialist visits and testing claims, and visits could have been unrelated to testing.

## Conclusions

In this cohort study of over 8000 rural Medicare beneficiaries diagnosed with advanced NSCLC from 2014 to 2019, molecular testing uptake was low despite guideline concordance and Medicare coverage. This study identified specific patient groups who were less likely to receive testing, which was associated with lower uptake of systemic therapy. The availability of resources to access and navigate complex subspecialty care may represent an important source of disparity in rural areas, highlighting areas for further study to support access to guideline-concordant care.

## Notes

Supplement 1. **eTable 1**. *ICD-O-3* Codes and Categories of Non–Small Cell Histology **eTable 2.** Health Care Common Procedure Coding System Codes Used to Identify Molecular Testing **eTable 3.** National Drug Codes Used to Identify Targeted Therapy Drugs **eTable 4.** Practitioner Specialty Codes and Categories Used to Categorize Practitioner Visits **eTable 5.** Descriptive Summary of Characteristics Measured at Non–Small Cell Lung Cancer Diagnosis, Among Those Continuously Enrolled in Part D for 6 Months After Diagnosis **eTable 6**. Cumulative Incidence of Molecular Testing by 180 Days After Diagnosis and Hazard Ratios for Differences in Time to Testing Between Groups, Among Those With Adenocarcinoma Histology Only (N = 3754) **eFigure. **Patient Subcohort Flowchart

Supplement 2.
Data Sharing Statement
