# Supplementary material for: Molecular Testing Among Patients With Advanced Non–Small Cell Lung Cancer in the Rural US
Source: JAMA Netw Open. 2026 Nov 5;9(11):e2642270. doi: 10.1001/jamanetworkopen.2026.42270 (PMC13741159; doi:10.1001/jamanetworkopen.2026.42270)
Supplement: Supplementary file 1 [file jamanetwopen-e2642270-s001.pdf]

Supplemental Online Content

Rhinehart D, van Gelderen E, Feliciano J, Pollack CE, Marrone KA, Blackford AL. Molecular testing among patients with advanced non–small cell lung cancer in the rural US. *JAMA Netw Open*. 2026;9(11):e2642270. doi:10.1001/jamanetworkopen.2026.42270

eTable 1. *ICD-O-3* Codes and Categories of Non–Small Cell Histology
eTable 2. Health Care Common Procedure Coding System Codes Used to Identify Molecular Testing
eTable 3. National Drug Codes Used to Identify Targeted Therapy Drugs
eTable 4. Practitioner Specialty Codes and Categories Used to Categorize Practitioner Visits
eTable 5. Descriptive Summary of Characteristics Measured at Non–Small Cell Lung Cancer Diagnosis, Among Those Continuously Enrolled in Part D for 6 Months After Diagnosis
eTable 6. Cumulative Incidence of Molecular Testing by 180 Days After Diagnosis and Hazard Ratios for Differences in Time to Testing Between Groups, Among Those With Adenocarcinoma Histology Only (N = 3754)
eFigure. Patient Subcohort Flowchart

This supplemental material has been provided by the authors to give readers additional information about their work.

© 2026 Rhinehart D et al. *JAMA Network Open*.

eTable 1. *ICD-O-3* Codes and Categories of Non–Small Cell Histology

ICD_O_3 Code	Description	Category
8040	Adenocarcinoma	Adenocarcinoma
8143	Superficial spreading adenocarcinoma	Adenocarcinoma
8140	Adenocarcinoma, not otherwise specified	Adenocarcinoma
8250	Lepidic adenocarcinoma	Adenocarcinoma
8252	Bronchiolo-alveolar carcinoma, non-mucinous	Adenocarcinoma
8253	Invasive mucinous adenocarcinoma	Adenocarcinoma
8254	Mixed invasive mucinous and non-mucinous adenocarcinoma	Adenocarcinoma
8255	Adenocarcinoma with mixed subtypes	Adenocarcinoma
8260	Papillary adenocarcinoma, NOS	Adenocarcinoma
8265	Micropapillary carcinoma, NOS	Adenocarcinoma
8323	Mixed cell adenocarcinoma	Adenocarcinoma
8480	Mucinous adenocarcinoma	Adenocarcinoma
8481	Mucin-producing adenocarcinoma	Adenocarcinoma
8570	Adenocarcinoma with squamous metaplasia	Adenocarcinoma
8574	Adenocarcinoma with neuroendocrine differentiation	Adenocarcinoma
8050	Papillary squamous	Squamous
8070	Squamous cell carcinoma	Squamous
8072	Squamous cell carcinoma, non-keratinizing	Squamous
8073	Squamous cell carcinoma, non-keratinizing	Squamous
8074	Pseudosarcomatous squamous cell carcinoma	Squamous
8083	Basaloid squamous cell carcinoma	Squamous
8084	Squamous cell carcinoma, clear cell type	Squamous
8094	Basosquamous carcinoma	Squamous
8123	Basaloid carcinoma	Squamous
8560	Adenosquamous carcinoma	Squamous
8000	Malignant neoplasm	Other/NOS
8001	Tumor cells, malignant	Other/NOS
8010	Carcinoma, not otherwise specified	Other/NOS
8012	Large cell carcinoma	Other/NOS
8013	Large cell neuroendocrine carcinoma	Other/NOS
8014	Large cell carcinoma with rhabdoid phenotype	Other/NOS
8020	Carcinoma, undifferentiated type, NOS	Other/NOS
8033	Pseudosarcomatous carcinoma	Other/NOS
8046	Non-small cell carcinoma	Other/NOS

© 2026 Rhinehart D et al. *JAMA Network Open*.

eTable 2. Health Care Common Procedure Coding System Codes Used to Identify Molecular Testing

Code	Description
81235	EGFR mutation
81400	Generic molecular pathology level 1
81401	Generic molecular pathology level 2
81402	Generic molecular pathology level 3
81403	Generic molecular pathology level 4
81404	Generic molecular pathology level 5
81405	Generic molecular pathology level 6
81406	Generic molecular pathology level 7
81407	Generic molecular pathology level 8
81408	Generic molecular pathology level 9
81415	Exome sequencing
81416	Comparator exome
81417	Exome reanalysis
81426	Comparator genome
81427	Genome reanalysis
81445	Solid tumor NGS panel 5-50 genes
81455	Solid tumor NGS panel 51+ genes
81479	Unlisted molecular pathology procedure
81504	Multianalyte Assays with Algorithmic Analyses
81540	Multianalyte Assays with Algorithmic Analyses
81599	Multianalyte Assays with Algorithmic Analyses
83890	Nucleic acid extraction
83891	Highly purified nucleic acid extraction
83892	Enzymatic digestion
83893	Blot production
83894	Gel electrophoresis
83896	Nucleic acid probe
83897	Nucleic acid transfer
83898	Target amplification
83900	Multiplex amplification
83901	Additional multiplex amplification
83902	Reverse transcription
83903	Mutation scanning
83904	Mutation identification by sequencing
83905	Allele-specific transcription
83906	Allele-specific translation
83907	Cell lysis prior to extraction
83908	Signal amplification
83909	High-resolution separation
83912	Molecular diagnostic interpretation and report
83913	RNA stabilization
83914	Primer extension / ligation assay
88271	FISH probe

© 2026 Rhinehart D et al. *JAMA Network Open*.

88272	FISH analysis 3-5 cells
88273	FISH analysis 10-30 cells
88274	FISH analysis 25-99 cells
88275	FISH analysis 100-300 cells
88291	Cytogenetic interpretation
88299	Unlisted cytogenetic study
88363	Examination and selection of retrieved archival tissue(s) for molecular analysis
88364	single-probe in situ hybridization (ISH)
88365	single-probe stain procedure for in situ hybridization (ISH)
88366	In situ hybridization (eg, FISH), per specimen; each multiplex probe stain procedure
88367	morphometric analysis performed via in situ hybridization
88368	morphometric analysis using in situ hybridization
88369	quantitative or semi-quantitative morphometric analysis using in situ hybridization
88373	morphometric analysis performed via in situ hybridization (ISH) using computer-assisted technology
88374	automated, computer-assisted morphometric analysis of in situ hybridization
88377	manual morphometric analysis using in situ hybridization (ISH) for a multiplex probe stain
88384	array-based evaluation of multiple molecular probes
88385	array-based evaluation of multiple molecular probes (51-250 probes)
88386	array-based evaluation of multiple molecular probes
0013U	proprietary genetic laboratory analyses
0019U	proprietary RNA laboratory test
0022U	proprietary NGS test
0036U	proprietary whole exome test
0048U	proprietary Integrated Mutation Profiling of Actionable Cancer Targets test
0057U	proprietary genetic laboratory analyses
0094U	proprietary whole genome sequencing test

© 2026 Rhinehart D et al. *JAMA Network Open*.

eTable 3. National Drug Codes Used to Identify Targeted Therapy Drugs

Class	Drug	National Drug Code
**EGFR**	Osimertinib	00310-1349-30
		00310-1350-30
	Erlotinib	72485-0217-30
		72485-0219-30
		00378-7132-93
		00378-7133-93
		00378-7131-93
		72485-0218-30
		63304-0096-30
		63304-0095-30
		00093-7664-56
		00093-7663-56
		54569-5848-00
		54569-5847-00
		50242-0062-01
		50242-0064-01
		50242-0063-01
		54868-5290-00
		54868-5447-00
		54868-5474-00
	Afatinib	00597-0141-30
		00597-0137-30
		00597-0138-30
	Dacomitinib	0069-0197-30
	Gefitinib	00310-0482-30
**ALK/ROS**	Alectinib	50242-0130-01
	Brigatinib	76189-0113-21
		76189-0113-18
		63020-0180-30
		63020-0090-30
		63020-0113-30
		63020-0090-07
		63020-0198-30
	Ceritinib	00078-0694-84
		00078-0640-70
	Crizotinib	00069-8140-20
		00069-8141-20
	Lorlatinib	00069-0231-01
		00069-0227-01
**BRAF**	Dabrafenib	00173-0847-08
		00078-0682-66
		00173-0846-08
		00078-0681-66
	Trametinib	00078-0668-15
		00173-0849-13
		00078-0666-15
		00173-0848-13
	Vemurafenib	50242-0090-02
		50242-0090-01

© 2026 Rhinehart D et al. *JAMA Network Open*.

**NTRK**	Entrectinib	50242-0091-30
		50242-0094-90
	Larotrectinib	50419-0391-01
		50419-0392-01
		71777-0391-01
		71777-0392-01
		71777-0390-01

© 2026 Rhinehart D et al. *JAMA Network Open*.

eTable 4. Practitioner Specialty Codes and Categories Used to Categorize Practitioner Visits

Outpatient Code	Carrier Code	Specialty	Category
2	3	General Surgery	Proceduralist
29	29	Pulmonary Disease	Proceduralist
33	33	Thoracic Surgery	Proceduralist
83	83	Hematology/Oncology	Oncologist
90	90	Medical Oncology	Oncologist
91	91	Surgical Oncology	Proceduralist
92	92	Radiation Oncology	Oncologist
94	94	Interventional Radiology	Proceduralist
78	NA	Cardiac Surgery	Proceduralist

© 2026 Rhinehart D et al. *JAMA Network Open*.

eTable 5. Descriptive Summary of Characteristics Measured at Non–Small Cell Lung Cancer Diagnosis, Among Those Continuously Enrolled in Part D for 6 Months After Diagnosis

**Characteristic**	**N=2046**
**Age (y)**	
Mean (SD)	74.9 (6.1)
Median (Range)	74.0 (66.0-94.8)
**Sex – participant no. (%)**	
Male	1043 (51.0)
Female	1003 (49.0)
**Ethnicity and Race – participant no. (%)**	
Hispanic (All Races)	65 (3.2)
NH AI/AN or API	28 (1.4)
NH Black	106 (5.2)
NH White	1847 (90.3)
**Marital Status – participant no. (%)**	
Married/Partner	721 (35.2)
Single, Divorced, Widowed	647 (31.6)
Unknown	678 (33.1)
**SEER Region – participant no. (%)**	
North East	232 (11.3)
South	1105 (54.0)
Midwest	302 (14.8)
West	407 (19.9)
**Year of Diagnosis – participant no. (%)**	
2014	381 (18.6)
2015	356 (17.4)
2016	311 (15.2)
2017	342 (16.7)
2018	322 (15.7)
2019	334 (16.3)
**Histologic Type – participant no. (%)**	
Adenocarcinoma	1099 (53.7)
Squamous	618 (30.2)
Other or NOS	329 (16.1)
**Charlson Comorbidity Index – participant no. (%)**	
0	1118 (54.6)
1	565 (27.6)
2+	363 (17.7)
**Hospitalized in year before diagnosis – participant no. (%)**	

© 2026 Rhinehart D et al. *JAMA Network Open*.

No	1636 (80.0)
Yes	410 (20.0)
**Frailty Score – participant no. (%)**	
< 0.15	1084 (53.0)
≥ 0.15	962 (47.0)
**Any Medicaid (Full or Partial) Eligibility at Diagnosis – participant no. (%)**	
No	1535 (75.0)
Full or Partial Medicaid	511 (25.0)
**Reason for Medicare Eligibility – participant no. (%)**	
Age and Survivor Insurance	1695 (82.8)
Disability and/or End stage renal disease	351 (17.2)
**Part D Low Income Cost Sharing – participant no. (%)**	
Full	536 (26.2)
Partial	91 (4.4)
Not Eligible	1419 (69.4)
**Yost Index State Quintile – participant no. (%)**	
1	5231(25.5)
2	780 (38.1)
3	545 (26.6)
4	169 (8.2)
5	31 (1.5)

Abbreviations: NH, Non-Hispanic; AI/AN, American Indian or Alaska Native; API, Asian or Pacific Islander; SEER, Surveillance, Epidemiology, and End Results; NOS, Not otherwise specified.

© 2026 Rhinehart D et al. *JAMA Network Open*.

eTable 6. Cumulative Incidence of Molecular Testing by 180 Days After Diagnosis and Hazard Ratios for Differences in Time to Testing Between Groups, Among Those With Adenocarcinoma Histology Only (N = 3754)

**Patient Subgroup**	**Cumulative incidence at 180 days**	**Hazard ratio**	**P-value**
**Age (y)**			
66-74	0.58 (0.56-0.61)	1.0	
75-84	0.57 (0.54-0.59)	0.96 (0.87-1.05)	0.34
84+	0.46 (0.41-0.51)	0.77 (0.66-0.91)	0.002
**Sex**			
Male	0.55 (0.53-0.57)	1.0	
Female	0.58 (0.56-0.61)	1.07 (0.98-1.16)	0.12
**Ethnicity and Race**			
Hispanic (All Races)	0.58 (0.49-0.67)	1.0 (ref)	
NH AI/AN or API	0.67 (0.54-0.81)	1.02 (0.67-1.54)	0.94
NH Black	0.49 (0.42-0.57)	0.75 (0.55-1.03)	0.08
NH White	0.57 (0.55-0.58)	0.90 (0.71-1.15)	0.40
**Marital Status**			
Married/Partner	0.59 (0.57-0.62)	1.0 (ref)	
Single, Divorced, Widowed	0.55 (0.52-0.58)	0.89 (0.80-0.99)	0.04
Unknown	0.55 (0.52-0.58)	0.93 (0.84-1.02)	0.13
**SEER Region**			
North East	0.57 (0.53-0.62)	1.0 (ref)	
South	0.52 (0.5-0.54)	0.90 (0.78-1.03)	0.13
Midwest	0.63 (0.59-0.67)	1.19 (1.01-1.41)	0.03
West	0.62 (0.59-0.66)	1.16 (1.00-1.35)	0.06
**Year of Diagnosis**			
2014-2016	0.55 (0.52-0.57)	1.0 (ref)	
2017-2019	0.59 (0.56-0.61)	1.06 (0.97-1.15)	0.22
**Charlson Comorbidity Index**			
0	0.57 (0.55-0.59)	1.0 (ref)	
1	0.59 (0.56-0.62)	1.06 (0.96-1.17)	0.24
2+	0.52 (0.48-0.56)	0.92 (0.81-1.04)	0.17
**Hospitalized in year prior to diagnosis**			
No	0.58 (0.56-0.6)	1.0 (ref)	
Yes	0.5 (0.46-0.53)	0.90 (0.80-1.00)	0.06
**Frailty index**			
<0.15 (not frail)	0.6 (0.58-0.62)	1.0 (ref)	
≥0.15 (frail)	0.53 (0.51-0.55)	0.90 (0.82-0.98)	0.01
**Any Medicaid (Full or Partial) Eligibility at Diagnosis**			
No	0.59 (0.58-0.61)	1.0 (ref)	
Full or Partial Medicaid	0.43 (0.39-0.47)	0.64 (0.56-0.73)	<0.001
**Reason for Medicare Eligibility**			
Age or Survivorship	0.58 (0.56-0.6)	1.0 (ref)	

© 2026 Rhinehart D et al. *JAMA Network Open*.

ESRD and/or Disability	0.48 (0.43-0.52)	0.78 (0.68-0.89)	<0.001
**Part D Low Income Cost Sharing**			
Not Eligible or Not Receiving Full Subsidy	0.59 (0.58-0.61)	1.0 (ref)	
Fully Subsidized	0.43 (0.4-0.47)	0.65 (0.57-0.73)	<0.001
**Yost Index State Quintile**			
1	61.1 (57.2-64.7)	1.0 (ref)	
2	66.0 (62.9-68.8)	1.12 (1.00-1.26)	0.05
3	68.5 (65.1-71.6)	1.24 (1.10-1.39)	<0.001
4 or 5	70.3 (64.1-75.4)	1.25 (1.07-1.47)	0.006

Abbreviations: NH, Non-Hispanic; AI/AN, American Indian or Alaska Native; API, Asian or Pacific Islander; SEER, Surveillance, Epidemiology, and End Results; ESRD, End-stage Renal Disease

© 2026 Rhinehart D et al. *JAMA Network Open*.

eFigure. Patient Subcohort Flowchart

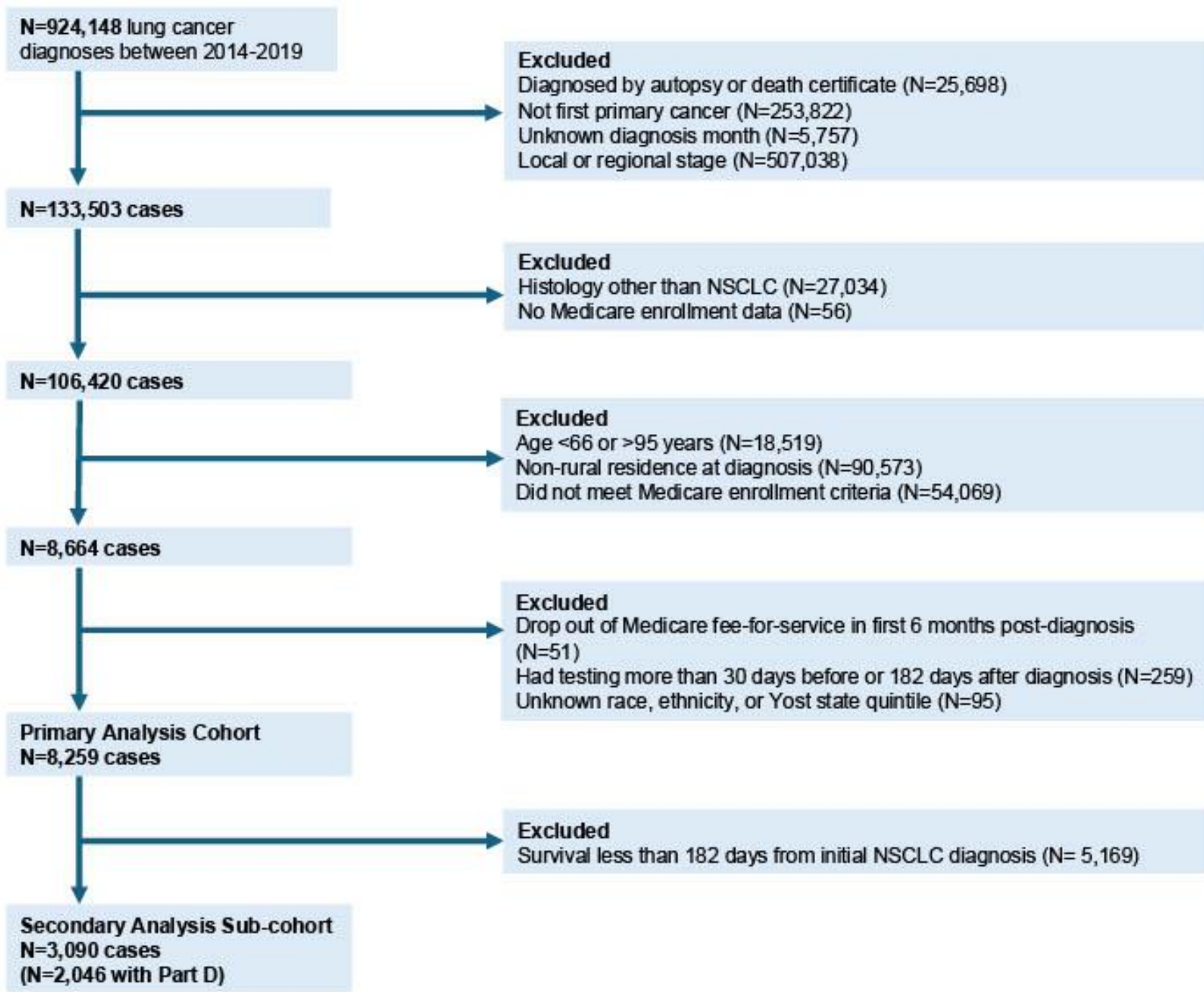

© 2026 Rhinehart D et al. *JAMA Network Open*.
