# Supplementary material for: Molecular Testing Among Patients With Advanced Non–Small Cell Lung Cancer in the Rural US
Source: JAMA Netw Open. 2026 Nov 5;9(11):e2642270. doi: 10.1001/jamanetworkopen.2026.42270 (PMC13741159; doi:10.1001/jamanetworkopen.2026.42270)
Supplement: Supplementary file 2 [file jamanetwopen-e2642270-s002.pdf]

Data Sharing Statement

Rhinehart. Molecular Testing Among Patients With Advanced Non–Small Cell Lung Cancer in the Rural US. *JAMA Netw Open*. Published November 05, 2026.
doi:10.1001/jamanetworkopen.2026.42270

Data
Data available: No

Additional Information
Explanation for why data not available: The dataset used to conduct this study is available upon approval of a research protocol from the National Cancer Institute. Instructions for obtaining these data are available at
https://healthcaredelivery.cancer.gov/seermedicare/obtain/.
